# Traumatic Extradural Hæmorrhage. Part I
*Reprinted by kind permission from the *British Medical Journal*, June 1st, 1940, p. 884.


**Published:** 1940

**Authors:** A. Rendle Short, Marjorie Dunster

**Affiliations:** Professor of Surgery in the University of Bristol


### TRAUMATIC EXTRADURAL HÆMORRHAGE.

### PART I.*

BY

A. RENDLE SHORT, M.D., B.S., B.Sc., F.R.C.S.,

*Professor of Surgery in the University of Bristol.*

AND

MARJORIE DUNSTER, M.B., CH.B.

---

As a general rule common diseases and accidents have been recognized and described for many years, and rarer conditions have been much more recently brought under the notice of the profession. To this rule there are some curious exceptions, and one of these concerns traumatic extradural hæmorrhage. Text-books of surgery fifty years old describe the well-known clinical picture: a brief period of concussion, perhaps only momentary, the lucid interval lasting a few minutes or hours, followed by renewed unconsciousness, with deepening signs of cerebral compression such as stertorous breathing, slow full hard pulse, fixed dilated pupils, hemiplegia, and so on. The trouble is due, of course, to a rupture of the middle meningeal artery, and the syndrome is important because timely operation may save the patient's life. As a matter of general experience, however, this clinical picture is very seldom seen. Only once in nearly forty years has one of us (A. R. S.) met with a typical case that was correctly diagnosed and recovered after operation. Middle meningeal hæmorrhage is not very uncommon in the post-mortem room, but in such cases the lucid interval was usually absent and the diagnosis made was cerebral compression of unknown character.

It is the purpose of this communication to describe a case giving the typical history of extradural hæmorrhage in which the bleeding vessel was not the middle meningeal artery; and a further case in which the middle meningeal artery was the source of the bleeding, the diagnosis of intracranial hæmorrhage and of its location was correctly made in the absence of the text-book signs, and a method of location was used which proved to be more accurate than the neurological findings.

* Reprinted by kind permission from the *British Medical Journal*, June 1st, 1940, p. 884.

**Case 1.**—A young French lady, aged 23, on a visit to England, was involved in a motor-car smash at midnight. Her head was injured, but she was able to walk and talk, and went into a house. After a few minutes she became unconscious, and was admitted to hospital about an hour and a half after the accident. When seen at 2 a.m. she was deeply comatose, with widely dilated pupils which did not react to light, Cheyne-Stokes respiration, and a pulse of 100, not of full volume. The scalp was bruised over the vertex and parietal regions. The right arm and leg were slightly spastic, and the left arm and leg markedly so, with exaggerated knee-jerk and extensor plantar response. The rapid advance of symptoms made it evident that there was a spreading intracranial hæmorrhage.

At 3 a.m. an operation was performed with novocain local anæsthesia. A small burr-hole exploratory opening was made over the right parietal region ; nothing was found except a fissured fracture of the skull. A similar opening was made on the left side. There was extensive blood clot spread out between the dura and the vertex of the skull, derived from the superior longitudinal sinus. The fissured fracture crossed the vertex transversely. The clot was cleared out. The patient died on the table shortly after the end of the operation.

The special point of interest in this case is that there was a typical history of a lucid interval lasting a few minutes, followed by unmistakable signs of compression, but the hæmorrhage was derived from the superior longitudinal sinus and not from the middle meningeal artery.

**Case 2.**—A working man aged 42 was admitted to hospital, having been knocked off his bicycle the previous afternoon. He was in a very restless unconscious condition from the time of the accident until three days after admission. He then became quiet and sensible, but had no recollection of the accident. After four days he became more restless, and wandered mentally at times. This state persisted for a few days, then he became quiet, but did not regain a completely rational outlook, being rather garrulous.

On admission the central nervous system showed no abnormal signs. There was slight bleeding from the right ear, which cleared up next day. A radiograph revealed a fissured fracture of the right parietal bone. The pulse rate was 84. From the fourth to the seventh day the patient complained of slight headache. The further progress of the case is as follows.

8*th Day.*—Patient restless ; intolerable headache. Central nervous system : pupils were equal, and responded to light and accommodation ; no nystagmus ; knee-jerk slightly increased on left side ; plantar response on left equivocal ; tone normal. Pulse rate 60. Lumbar puncture : greatly increased pressure, roughly estimated at 600 mm. water ; fluid was yellow.

9*th Day.*—Patient complained of severe headache, and wandered mentally. Central nervous system as on eighth day. Lumbar puncture: pressure 600 mm. ; 60 c.cm. slowly withdrawn.

10*th Day.*—Patient quieter. Ankle-jerk and knee-jerk increased on left side ; plantar response on left side extensor ; tone equal. Lumbar puncture : pressure 350 mm. ; 60 c.cm. withdrawn.

*11th Day.*—Headache much relieved. Ankle-jerk and knee-jerk increased on left ; plantar response still extensor ; ankle clonus present ; tone slightly raised ; abdominal reflexes all present ; no anæsthesia ; no astereognosis ; no nystagmus ; pupils normal ; no facial weakness ; papilloedema pronounced. Lumbar puncture : pressure 100 mm. Patient continued with no change.

*16th Day.*—Electro-encephalogram taken. Mr. W. Grey Walter of the Burden Neurological Institute reported as follows : " Electro-encephalograms were taken at the Burden Neurological Institute on November 25, 1939. Records from the left side were entirely normal (Fig. A, p. 87). The superior part of the right hemisphere was also normal, but low down on the right side a prominent and well-defined delta focus was found (see Fig. B). The first few delta waves in the record shown are shaded to emphasize the phase relations of the waves, since it is upon these that the localization is based. Such a slow delta discharge indicates an abnormal area of cortex, but does not give information as to its cause. It is most common in cases of cerebral tumour (Walter, 1937), but comparatively few cases of head injury have been examined, though it is known that in recovered cases the record is normal. The findings in this case suggested that something equivalent to a cerebral neoplasm had arisen in the area shaded in the figure."

*19th Day.*—One of us (A. R. S.) operated for removal of extradural hæmatoma. An extradural blood clot about the size of the palm of the hand was found at the site indicated by the electro-encephalogram, over the lower Rolandic area. A small trephine-hole was made over the lower part of the Rolandic area and an extradural clot was seen. An osteoplastic flap with a radius of two and a half inches from the first trephine-hole was turned back. The clot was removed. A posterior branch of the middle meningeal artery was found to be bleeding. This was secured by suture and covered with Horsley's wax. The flap was replaced and the scalp sutured.

After operation a blood transfusion of two pints was given. The patient progressed favourably, and was discharged after four weeks, his mental condition being normal. The central nervous system was normal except for a slight increase in the knee-jerk on the left side.

### DISCUSSION.

This case history presents a number of points of special interest. In the first place, the clinical picture was quite unlike the text-book description of extradural hæmorrhage. There was no lucid interval, and the signs of pressure were psychical rather than physical. The diagnosis was not made until the third week after the injury. In the second place, the neurological signs gave a false location and the electro-encephalogram a true one. Thirdly, the value of a late decompression operation was demonstrated. No doubt the patient was fortunate in that the bleeding ceased before the pressure was raised so high as to be fatal. The evidences of raised pressure were : (*a*) the mental symptoms ; (*b*) the exaggerated knee-jerk and extensor plantar response on the left side ; (*c*) papilloedema ; (*d*) the high pressure of the cerebrospinal fluid. The fact that the fluid was lemon-coloured suggested that the amount of blood that had reached it was inconsiderable, which pointed to extradural rather than to intradural hæmorrhage.

Although the condition is not often recognized, at any rate by the majority of surgeons, there are good reasons for considering that persistent rise of intracranial pressure as a result of extradural or intradural bleeding is not very uncommon. In his classical monograph in Choyce's *System of Surgery* the late Wilfred Trotter has a section on the subject of latent subdural hæmorrhage, in which he says that these cases may run a course of several weeks or even two or three months. There is a long prodromal period in which the symptoms are " slight, ambiguous, or misleading, and a short period during which serious symptoms rapidly develop and the presence of a definite wide-spread compression becomes manifest. The accident may be severe, slight, or even apparently trivial." The symptoms during the prodromal period are headache and giddiness in many cases, but mental changes are the commonest. " Changes of disposition, attacks of irritability, lethargy, and loss of initiative are amongst the slight manifestations. There may also be transient attacks of mental confusion or of unconsciousness, possibly so slight as not to arouse serious attention. Persistent drowsiness, with perhaps mild delirium at night, occurs in some cases. It is unusual for definite physical signs of compression to be present, but such may come and go in a very surprising and puzzling way. At one examination an alteration of reflexes of hemiplegic type may be found, while the next day no such abnormality can be discovered. Often a fullness of the retinal veins on one side may be found, and as a rarity definite optic neuritis."

At the end of the prodromal period the headache may suddenly increase, vomiting occur, the drowsiness deepen to coma, and hemiplegia develop. The hæmorrhage is usually to be found over the lateral part of the cerebral hemisphere.

Wilfred Trotter does not describe similar symptoms due to extradural hæmorrhage as in the present case.

### The Literature.

A number of papers have appeared in recent American surgical literature dealing with chronic subdural hæmatoma. The most interesting of these is by two Boston neurosurgeons, G. Horrax and J. L. Poppen (1937), who report as many as eighteen cases seen within the space of two years. In some of these the history of injury was trivial and the patient was sent up with a suggested diagnosis of cerebral tumour, arteriosclerosis of the vessels of the brain, or " post-encephalitic state." The symptoms were headache, mental changes, or lapses into drowsiness or coma. These surgeons made the diagnosis by means of exploratory burr-holes over the posterior parieto-occipital regions on both sides, about 4 cm. from the middle line. If a subdural hæmatoma is present it shows blue-black through the dura. The usual situation of these hæmatomata is over the upper parietal region of the brain. They are as a rule sufficiently fluid to be washed out by means of a catheter. A perfect functional result is generally obtained. These authors do not refer to any cases of chronic extradural hæmatoma, but it is evident that the symptoms may be very similar. If in our second case the burr-hole exploration had been made in the situation advocated by Horrax and Poppen the haematoma would have been missed.

In this country Professor Lambert Rogers (1937), like the American writers, has laid stress on the importance and frequency of chronic symptoms, due to intracranial hæmorrhage following a head injury. In four cases described by him the operation was performed three days, five days, twenty-two days and three months, respectively, after the accident. In the first and second cases the hæmorrhage was extradural and derived from the middle meningeal artery; in the third and fourth it was subdural. In the first case the indication for operation was the development of hemiplegia in a patient who had never recovered from unconsciousness with cerebral irritation. In the second case the patient did not lose consciousness until the fifth day, but complained of headache. The third case had headache for a fortnight and then became drowsy and hemiplegic. The fourth case was suspected of a frontal cortical tumour.

### Location by Electro-encephalography.

Of perhaps even greater interest than the occurrence of latent extradural hæmorrhage is the method of location by means of electro-encephalograms. The necessary apparatus has recently been made available in Bristol at the Burden Neurological Clinic under the direction of Professor Golla. The great advantage of the method is its relative simplicity and harmlessness—quite unlike ventriculography and encephalography, which are not simple, nor free from all risk of unpleasant consequences. It remains for the future to show how accurate location by this method will prove to be. In the case under consideration the only evidence as to the site of the hæmorrhage was given by the fissured fracture of the skull and by the neurological findings—that is to say, the exaggerated knee-jerk and extensor response on the left side. This might have led us to trephine over the leg area. If this had been done the clot would have been completely missed. The fracture, on the other hand, was near the actual lesion. Trephining over the silent area indicated by the electro-encephalogram brought us down upon the very centre of the extradural hæmorrhage. It is very clear from these records and observations that when persistent headache or mental changes follow a head injury and do not resolve for days or weeks the possibility of an extradural or subdural hæmatoma should be carefully considered and steps be taken to investigate the matter. In particular lumbar puncture and electro-encephalography would appear to be indicated. If information can be obtained in no other way exploration by burr-holes after the method of Horrax and Poppen might be justified. Missed cases will probably suffer from persistent headache and mental symptoms, or there may be a late oncoming of monoplegia or hemiplegia. Cases correctly diagnosed can be relieved of all their symptoms by operation.

REFERENCES.

Horrax, G., and Poppen, J. L. (1937), *New Engl. J. Med.*, 216, 381.

Rogers, Lambert (1937), *J. Internat. Chir.*, 11, No. 2.

Walter, W. Grey (1937), *Proc. Roy. Soc. Med.*, 30, 579.

